# Supplementary material for: Degenerate sequence-based CRISPR diagnostic for Crimean–Congo hemorrhagic fever virus
Source: PLoS Negl Trop Dis. 2022 Mar 10;16(3):e0010285. doi: 10.1371/journal.pntd.0010285 (PMC8939784; doi:10.1371/journal.pntd.0010285)
Supplement: S1 File — This file contains references [63,64]. Fig A. DNA templates for in vitro transcription of the S segment from CCHFVs. Fig B. DNA templates for in vitro transcription of the S segment from CCHFV-related viruses. Fig C. Shannon entropy plot of 265 CCHFV isolates. Fig D. The degenerate sequence-based CRISPR diagnostic generates significant amplification signals rapidly at 1 cp/μl of CCHFV RNA from any clade/sub-clade. Fig E. Significant signal amplification with CCHFV but not closely related viruses. Fig F. Human genomic DNA contamination does not lead to false positive signals in the degenerate sequence-based CRISPR diagnostic for CCHFV. Fig G. Clade location of the IND and UAE isolates. (PDF) [file pntd.0010285.s001.pdf]

>1.ArD (Insert sequence below contains BglII_T7(+)_DQ211640(-)_BamHI_SP6(-)_XbaI, was cloned into pET-28c(+) between BglII and XbaI, and finally linearized with BglII and BamHI)
AGATCTCGATCCCGCGAAATTAATACGACTCACTATAGGGTCTCAAAGATATCGTTGCCGCACAGCCCTTTAATATGTAAAAGAAGAATATAATAGTATGAAGCAAAATAGAATAAAGCAAGATAAAATAGGTTTTGGTAACTGCAAAAAGCAAATGCGATTAATGGTTATGGTCAGAA
GCAGAAGCACAAACAGGAACAAATAGAAAAAAACTCTGGAGATTTAGATAATGTTGGCACTGGTGGCATTGCCTTTGACGTTGTAAGCATTTTGGAACGGGGATTGCTTGCCAACAAGAGACTGGTGCAGCAGGTGCTCAGAGGCAACAATATCCATGTCCTGTATGTTGAATCCTGTT
TTTTGAACTTCGAAAAGTTTGACAATCGTTTTAGCACAAGGATTGTTAGTTTCACTATTTGTTCGGAGGTTCAGAATTGACTTGGTGTGACCTGAGCCCTGGGCTGCATCGTCTGGATTGGCAACAGGGATGGTTCCAAAGCAAACACCCATCTCACTAATTCTGCCAGCTGTCAGCAC
AGCAGGGTGCATGTAGATCCTGTTTTGCTGGAAAGAGTCATCAGCGAAGAGCTCATAAAGCTTCTTTCCCCATTTCATTGGGGTGCTCAGAAGTGCCTTCTTCATTTTTTTAGTGCCTCTTGGCTGCTTTCCCAGCTCAAAGAGGAATTGAGAGACGGTGGGGAAGGTTTCTGGAGTCA
CGCCCGCTTTATAGAGCCAGTAGAAGGAGCTGAAAGGGGTGTCAATCTGTGCACCCTGTGCTCTCAGTGCTGATGAGTTTTTGTAGAGTTCCTGGGCCTTAGTGATGTGCTTGAGGAGGTTTGTTACCATGCTGTCAGCACTAGCCTTGTCCACCTCATCTTTGTGTTTGTCAAGGTAC
TCCTTGAGGGCCTCCACTGTCTTCTTGGCCTCCTCAAAAACTCCCTTTCCTTCTGTTTCTGCAAGTTTGGCAAGACCTGTAGCAACTAGAGCTATTCCTGAGCGGCCGGACTTGTTGACATCACCCCAGGGTGGATTGAAGGCCATTATATATTTGCCCTTGACAAACTCCCTGCACCA
TTCTATGTGCTCCTTGCTTACTGGGCCTCTTGGGTTCTCATCACCTCCTCTGTTAAGGATCAGGTTCCTCCTTCTAATCATGTCTGAAAGCATCTCTTTGACAGACATCACAATCTCACCAGGAACCTTGTACTCAGAGAGGACTTTGTTGCTTAGGGCCGCTGTGTTTGCATTAACGC
GGAAGCCTATGTCCTTCCTCCACTTGAGGGCGGCCTGCTGATAGTTGGCAAGCTGTTCAATTTTGGGCACATCCACTTTCAGCTCAGTATAGCTCTCATCCCAAGACTTAATGGTTCCTGTGTTTTTTTCAAACCACTCAAGGCCTCTCTTCACTATACCTGTGGAGCTAACCCAGGCA
CATTCATATATGGGAGCACAGTACTTGGTGGCCTCAACCAGGGCAGATGCATAGATGGAGTCTTTTTGTGCATCATCGGTTGCACCAGCCATCTGGAATACAAACCTGTCAAGGTTCGGCACGTTCTCACAGAAAGAATAGGAGTTTGTGAAAGTATCCACAAGCCCGTTTCCTTTCTT
GAACTCCTCAAACCATTTGTTCAGCTCATCTTTGTTGTTCACTTCGATTTTGTTCTCCATTTTGCTAACACTCAAGGTAACACTGTGGGCGTAAGCGGCACGTGTTTCTTTGAGAGGATCCTTCTATAGTGTCACCTAAATGCGCTCTAGA

>2.UG (Insert sequence below contains BglII_T7(+)_DQ211650(-)_BamHI_SP6(-)_XbaI, was cloned into pET-28c(+) between BglII and XbaI, and finally linearized with BglII and BamHI)
AGATCTCGATCCCGCGAAATTAATACGACTCACTATAGGGTCTCAAAGATATCGTTGCCGCACAGCCCTTTAAGTGTTTAAATTAAGAAAAAAAGCAAAGTTAGTGAAGCTGGATTAAATAAACGATTGATAAAAGCAATTAGAGGAAAGAATCTGAGTGCAGAATGACACAAGTTTCA
GATAATGTTGGCACTGGTGGCGTTGCCTTTGACATTGTAGGCATTCTGGAAAGGAGACTGCTTTCCAACAAGAGACTGATGCAGCAGATGTTCAGAGGCCACAATATCCATATCCTGTATATTGAATCCTGTCTTTTGGATTTCAAACAGTTTAACAATTGTCTTAGCACATGGATTGT
TAGTCTCTGTGTTTGTCCGAAGGTTGAGAATGGACTTGGTGTGTCCTGATCCCTGGGCAGCGTCATCAGGGTTAGCCACGGGGATGGTTCCGAAGCAGACCCCCATCTCACTGATTCTGCCGGCTGTCAACACAGCAGGATGCATATAGATCCTGTTCTCCTGGAAAGAATCATCTGCA
AAGAGTTCATAGAGTTTCTTTCCCCACTTCATTGGAGTGCTCAGGAGTGCCTTCTTCATTTTCTTGGTGCCCCTTGGTTGCTTCCCCAGCTCAAAGAGAAACTGTGAAACAGTTGGGAAGGTTTCTGGAGATACGCCAGCCTTGTAAAGCCAGTAAAATGAGCTGAAAGGAGTGTCAAT
CTGTGCGCCTTGTGCACGGAGTGCAGATGAATTTTTGTAGAGCTCTTGTGCCTTTGCAATGTGCTTGAGGAGGTTTGTCACCATGCCGTCAGCACTGGCCTTGTCAACCTCATCCTTGTGCTTATCCAAATACCCATTAAGGTCCTCAACAGTTTTTTGGCTTCATCAAACACTCCTT
TCCCCTCAGTCTCTGCAAGCTTGGCAAGACCTGTTGCGACAAGAGCTATCCCCGAGCGGCCTGACTTGTTAATATCTCCCCAGGGTGGGTTGAAAGCCATGATGTACTTGCCCTTGACAAATTCCCTGCACCATTCCACATGCTCACGGCTCACCGGGCCGCGTGGGTTCTCATCGCCA
CCTCTGTTGAGGATCAAGTTCCTTCTTCTAATCATGTCAGACAACATCTCTTTGACAGACATTACAATCTCACCAGGGACTTTATACTCTGCAAGGACTTTGTTGCTCAAGGCTGCCGTATTAGCATTGACACGGAAACCAATATCCTTCCTCCACTTCAGAGCAGCCTGCTGGTAGTT
TGCAAGCTGCTCTATCTTGGGGACCCCAACTTTAAGTTCAGTATAGCTCTCATCCCAAGACTTGATGGTTCCTGCATTTTTTTCGAACCACTCAAGGCCTTTCTTGACTATGCCGGTGGAGCTGACCCATGCACACTCGTATATAGGTGCACAAAACTTGGTTGCCTCTACTAGAGCAG
ATGCATAGATGGAGTCCTTTTGTGCATCGTCTGTGGCACTGGCCATCTGAAACACAAACCTGTCAAGGTTAGGGACACTCTCACAAAAGGAGTAAGAGTTTGTGAAGGTGTCCACAAGTCCATTACTCTTCTTGAACTCTTCAAACCACCTGTTCATCTCATCCTTGCTGGTCACCTCG
ATTTTATTCTCCATTTTGCCAACACTCAAGTGAACACTGTGGGCGTAAGCGGCACGTGTTTCTTTGAGAGGATCCTTCTATAGTGTCACCTAAATGCGCTCTAGA

>3.IbAr (Insert sequence below contains BglII_T7(+)_U88410(-)_BamHI_SP6(-)_XbaI, was cloned into pET-28c(+) between BglII and XbaI, and finally linearized with BglII and BamHI)
AGATCTCGATCCCGCGAAATTAATACGACTCACTATAGGGTCTCAAAGATACCGTTGCCGCACAGCCCTTTAAGTGTTTAAAACAAGAGAAAATAGAGAAAGGTTAATAAAATAAGGTTACATAAGCAAGTAAAAAAGCATGATTAAGTATTGCAATTATGGATGGCGGTTCAGAAGGA
AGGAAGTTGAGTACAGAGCAGTTTGTATTTTAAATGATGTTAGCACTGGTGGCATTGCCCTTGACGTTGTAGGCGTTCTGGAATGGGGATTGCTTGCCAACAAGGGATTGGTGTAGCAAGTGCTCAGAGGCCACTATGTCCATGTCCTGAATGTTGAACCCTGTTTTTTGAACTTCAAA
TAGCTTGACGATGGTTTTGGCACACGGATTATTGGTCTCAGTGTTGGTACGGAGGTTGAGAATAGACTTAGTGTGTCCAGATCCTTGGGCAGCATCATCAGGATTGGCCACAGGGATTGTCCCAAAGCAGACTCCCATTTCACTGATTCTACCAGCTGTAAGCACGGCAGGATGCATGT
AAATCCTGTTCTGCTGGAAAGAATCATCGGCAAAGAGCTCATAAAGCTTCTTCCCCCACTTCATTGGGGTGCTCAGAAGAGCCTTCTTCATTTTCTTGGTACCTCTTGGCTGTTTCCCTAGCTCAAAGAGGAACTGTGACACCGTCGGGAAGGTTTCAGGAGTCACGCCAGCCTTGTAA
AGCCAATAGTATGAGCTGAAAGCAGTGTCAATCTGTGCGCTTTGTGCACGAAGTGCAGATGAATTTTTATAGAGCTCCTGTGCCTTGGCAATATGCTTAAGAAGGTTTGTTATCATGCTGTCGGCGCTTGCTCTATCAACTTCGTCCTTATGCTTGTCCAGATACCCGTTGAGGGCCTC
CACAGTCTTTTTGGCTTCATCAAATATTCCCTTTCCTTCAGTCTCTGCAAGCTTAGCAAGGCCTGTTGCAACAAGTGCTATTCCTGAACGGCCTGACTTGTTGATGTCCCCCCATGGTGGGTTGAAGGCCATGATGTATTTGCCTTTGACAAACTCCCTGCACCAGTCTACATGCTCAT
GGCTCACTGGGCCACGTGGGTTCTCATCACCACCCCTGTTTAGAATCAGGTTCCTTCTCCTAATCATGTCTGACAGCATCTCTTTGACAGACATCACAATCTCACCAGGGACTTTGTATTCTGCGAGGACTTTGTTGCTCAGAGCTGCTGTGTTGGCATTGACACGGAAACCTATGTCT
TTTCTCCACTTCAAGGCAGCTTGTTGGTAACCGGTAAGCTGCTCTATTTTCGGGACGTCGACCTTTAGCTCAGTATAACTTTCATCCCAGGACTTAATGGTTCCTGCATTTTTCTCGAACCATTCAAGTCCCTTTTTTACAATGCCAGTGGAGCTAACCCATGCGCACTCATATATAGG
TGCACAAAACTTTGTTGCCTCCACCAGAGCAGATGCGTAGATGGAGTCCTTCTGTGCATCATCGGTGGCACTGGCCATCTGAAACACAAACCTGTCCAAATTGGGAACACTCTCGCAAAAGGAATAGGAGTTTGTGAAGGTGTCCACAAGTCCATTTCCTTTTTTGAACTCTTCAAACC
ACCTGTTCATCTCATCTTTGTTATTCACCTCGATCTTGTTTTCCATTCTGCTAACACTCAAGAGAACACTGTGGGCGTAAGCGGCACGTGTTTCTTTGAGAGGATCCTTCTATAGTGTCACCTAAATGCGCTCTAGA

>4.1Bagh (Insert sequence below contains BglII_T7(+)_AJ538196(-)_BamHI_SP6(-)_NdeI, was cloned into pET-28c(+) between BglII and NdeI, and finally linearized with BglII and BamHI)
AGATCTCGATCCCGCGAAATTAATACGACTCACTATAGGGTCTCAAAGATATCGTTGCCGCACAGCCCTTTAAGTGTTTAAAACAGAAGAAAATAAGAAGATATTAATAAAGCAGGATTACATAAGCAATTAAAATAAACATGGTTGATGTTTGCAGTTGTGAACAGCGGAGCAAGAAT
AGGAAAGCTGTCTGCAGAGCATTCTGAACTTTAGATGATGTTGGCGCTGGTGGCATTCCCCTTGACGTTGTAGGCATTTTGAAATGGAGACTGCTTGCCAACAAGGGATTGGTGCAGTAGGTGCTCAGAGGCCACAATGTCCATGTGCTGTATGTTAAACCCTGTTTTCTGAATTTCAA
ACAACTTAACAATTGTTCTGGCACATGGATTGTTGGTCTCAGTGTTTGTCCGAAGGTTGAGAATGGACTTGGTGTGTCCAGATCCTAAGGCAGCATCATCAGGATTGGCCACCGGGATTGTTCCAAAGCAGACACCCATTTCACTGATTCTTCCGGCTGTCAGCACAGCGGGATGCATG
TAGATCCTGTTCTGCTGGAAAGAATCATCGGCAAAAAGCTCATAAAGCTTCTTTCCCCACTTCATTGGGGTACTCAGGAGTGCCTTCTTCATTTTCTTGGTACCCCTTGGATGCTTCCCTAACTCGAAAAGGAACTGTGAGACAGTTGGGAAGGTTTCTGGAGTCACACCAGCCTTGTA
AAGCCAGTAGTATGAGCTGAAGACAGTATCAATCTGTGCACCCTGTGCACGAAGAGCAGAAGAGTTTTTGTAAAGCTCTTGTGCCTTAGCGACATGCTTAAGAAGGTTTGTTACCATGTTGTCGGCACTTGCTTTGTCAACTTCGTCCTTGTGCTTGTCTAGATACCCGTTGAGAGCTT
CCACAGTCTTCTTGGCTTCGTCAAAGACTCCTTTCCCTTCAGTCTCTGCAAGCTTGGCAAGGCCTGTTGCAACAAGTGCTATTCCAGAACGGCCTGACTTGTTGATGTCCCCCCAAGGTGGGTTGAAAGCCATTATGTACTTGCCTTTGACAAATTCCCTACACCACTCCACATGTTCA
CGGCTGACTGGGCCGCGTGGGTTCTCGTCACCACCTCTGTTGAGGATCAAGTTCCTCCTTCTAATCATATCTGACAACATCTCTTTAACAGACATTACAATTTCGCCAGGAACTTTGTATTCTGCTAGGACTTTGTTACTTAAAGCTGCCGTGTTTGCATTGACACGGAAACCTATGTC
TTTCCTCCACTTGAGAGCAGCCTGCTGGTAGTTGAAAAGTTGTTCTATTTTGGGAACTTCAACTTTCAGCTCAGTATAACTCTCATCCCAAGATTTAATAGTTCCTGTATTTTTCTCAAACCACTCCAGTCCCTTCTTGACAATGCCAGTGGAGCTAGCCCAAGCACACTCGTATATAG
GTGCACAAAATTTGGTTGCTTCCACCAGAGCAGATGCGTAGATAGAGTCCTTTTGAGCATCATCAGTGGCACCAGCCATCTGAAATACAAATCTATCCAGATTTGGAACACTTTCACAAAAAGAATAGGAGTTTGTGTAAGTGTCCACAAGTCCATTCCCCTTCTTGAACTCCTCAAAC
CACTTGTTCATTTCATCCTTACTGTTCACTTCGATTTTGTTTTCCATTTTGCTAACACTCAAGAGAACACTGTGGGCGTAAGCGGCACGTGTTTCTTTGAGAGGATCCTTCTATAGTGTCACCTAAATGCGCCATATG

>4.2CHN (Insert sequence below contains BglII_T7(+)_AJ010649(-)_BamHI_SP6(-)_XbaI, was cloned into pET-28c(+) between BglII and XbaI, and finally linearized with BglII and BamHI)
AGATCTCGATCCCGCGAAATTAATACGACTCACTATAGGGTCTCAAAGATATCGTTGCCGCACAGCCCTTTAAGTGTTTAAACGAAAGAAACAAGAAGAAGTTAATAAAACAGGATTATATAAGCAGTTAAAATAAACATTGTTGATGGTTACAATCATAGATAGCAATGCAAAAGGGA
GGAAAGCAGAATGCAAAGCATTCGGAGCTTCAGATGATGTTGGCACTGGTGGCATTGCCCTTGACGTTGTAGGCATTCTGGAATGGAGACTGCTTGCCGACAAGGGATTGGTGCAGCAGGTGCTCAGAGGCTACAATGTCCATGTCTTGTATATTAAATCCTGTTTTTTGAATTTCAAA
CAACTTGACAATTGTCTTGGCACACGGATTGTTGGTTTCGGTGTTTGTCCGTAGGTTAAGAATGGACTTGGTGTGTCCAGATCCCTGGGCAGCATCATCGGGATTGGCCACAGGGATTGTTCCAAAGCAGACACCCATTTCACTGATTCTGCCAGCCGTCAACACGGCAGGGTGCATGT
AGATCCTGTTTTGCTGGAATGAGTCATCAGCAAAGAGCTCATAAAGCTTCTTACCCCACTTCATTGGGGTGCTCAAAAGTGCCTTCTTCATTTTCTTGGTACCCCTTGGTTGTTTCCCTAGCTCAAAAAGGAACTGTGAGACAGTCGGGAAGGTTTCTGGAGTCACGCCGGCCTTGTAG
AGCCAGTAGTATGAGCTGAAAGCAGTGTCAATCTGTGCACCCTGTGCACCAAGTGCAGACAAGTTTTTGTAAAGCTCTTGTGCCTTAGCAATGTGTTTGAAAAGGTTTGTTATCATGTTGTCGGCACTTGCTTTGTCAACTTCGTCCTTGTGTTTGTCAAGGTACCCGTTGAGAGCCTC
TACAGTCTTTTTGGCTTCATCAAACACTCCCTTCCCCTCAGTCTCTGCGAGCTTGGCAAGGCCTGTTGCAACAAGTGCTATTCCTGATCGGCCTGACTTGTTGATGTCCCCCCAGGGCGGGTTGAAAGCCATGATGTACTTGCCTTTGACAAATTCCCTGCACCACTCCACATGTTCAC
GGCTCACTGGGCCTCGTGGATTTTCATCGCCACCTCTGTTGAGAATCAAGTTCCTTCTTCTGATCATATCTGACAACATTTCTTTGACGGACATTACAATTTCGCCAGGGACTTTGTACTCTGCAAGGACCTTGTGGCTTAAGGCTGCCGTATTTGCATTGACACGAAAACCTATGTCC
TTCCTCCACTTGAGAGCAGCCTGTTGGTAATTGGCAAGCTGTTCTATTTAGGGACCTCAACTTTCAGCTCAGTGTAGCTTTCATCCCAAGATTTAATGGTTCCTGCATTCTTTTCGAACCACTCCAGTCCCTTCTTCACAATGCCAGTGGAGCTAACCCAGGCACACTCGTATATGGG
TGCACAAAATTTGGTTGCTTCCACTAGAGCTGATGCGTAGATGGAATCCTTTTGTGCGTCATCGGTGGCACTGGCCATCTGAAACACAAACCTTTCCAGATTTGGAACACTCTCACAAAAGGAGTAGGAGTTTGTGAAAGTATCCACAAGTCCATTTCCTTTCTTGAACTCTTCAAACC
ACTTGTTCATTTCATCTTTGTTATTCACCTCGATTTTATTCTCCATTTTGCTAGCACTCAAGAGAACACTGTGGGCGTAAGCGGCACGTGTTTCTTTGAGAGGATCCTTCTATAGTGTCACCTAAATGCGCTCTAGA

>5.Hoti (Insert sequence below contains BglII_T7(+)_DQ133507(-)_BamHI_SP6(-)_NdeI, was cloned into pET-28c(+) between BglII and NdeI, and finally linearized with BglII and BamHI)
AGATCTCGATCCCGCGAAATTAATACGACTCACTATAGGGTCTCAAAGATATCGTTGCCGCACAGCCCTTTAAGTATTTGAAATGAAAGATAGCAAGATAAAATTAGCAAAACAGCATTATATAAGCAATTAAAATAAACGTGATTAATAGTCATAACTGTAAGTAGTGATGCAGGAGG
AGAAAAGCTGAATGTGAAACGCCTTGAGTTCTAGATGATGTTGGCACTAGTGGCATTGCCCTTGACATTGTAGGCATTTTGGAATGGAGACTGCTTGCCAACAAGGGATTGGTGCAGCAGGTGCTCAGAGGCTACAATGTCCATGTCCTGTATGTTAAATCCTGTTTTTTGGATTTCAA
ATAATTTGACAATTGTCTTGGCACATGGGTTGTTGGTCTCTGTGCTTGTCCGAAGGTTGAGAATGGACTTGGTATGTCCAGATCCCTGAGCAGCGTCATCAGGATTGGCAACAGGGATTGTTCCAAAGCAGACACCCATTTCACTGATTCTACCGGCTGTCAACACAGCAGGGTGCATG
TAGATTCTGTTCTGCTGGAAAGAGTCATCAGCAAAGAGCTCATAAAGCTTCTTCCCCCACTTCATTGGAGTGCTCAGGAGTGCCTTTTTCATTTTTTTGGTCCCCCTTGGCTGCTTCCCCAGTTCGAAGAGGAACTGTGAGACAGTTGGGAAGGTCTCTGGAGTCACACCGGCCTTGTA
GAGCCAGTAAAACGAGCTGAAAGGAGTGTCAATCTGTGCACCTTGTGCACGAAGAGCAGATGAATTTTTATAAAGCTCTTGTGCTTTGGCAATGTGCTTTAGGAGGTTTGTTATCATGCTGTCGGCACTTGCTTTGTCAACTTCGTCCCTGTGCTTGTCCAAATACCCATTGAGAGCCT
CCACGGTCTTCTTTGCTTCGTCAAAGACTCCTTTCCCCTCGGTCTCTGCAAGCTTGGCAAGGCCTGTTGCAACAAGTGCTATTCCTGAACGGCCTGATTTGTTGATGTCCCCCCAAGGTGGATTGAAGGCCATGATGTACTTGCCTTTGACAAACTCCCTGCACCACTCCACATGTTCA
CGGCTCACTGGGCCGCGTGGATTTTCATCACCGCCCCTGTTGAGAATTAGATTCCTCCTTCTAATCATGTCTGACAGCATTTCTTTAACAGACATCACAATTTCGCCAGGGACTTTATATTCTGCAAGGACTTTGTTGCTTAAGGCTGCCGTGTTTGCATTGACACGGAAACCTATGTC
CTTCCTCCACTTGAGAGCAGCCTGTTGGTAATTGGCAAGTTGTTCTATTTTGGGAACATCAACCTTCAGCTCAGCATAGTTCTCATCCCAAGATTTGATGGTTCCTGAATTCTTCTCAAACCACTCAAGCCCCTTCTTCACAATGCCAGTAGAGCTGACCCAAGCACATTCATATATGG
GTGCACAGAACTTGGTTGCTTCCACCAGAGCCGATGCATAGATGGAGTCCTTCTGTGCATCATCAGTGGCGCTGGCCATCTGGAACACAAACTTATCCAGATTTGGTACATTCTCGCAAAAGGAGTAGGAGTTTGTGAAAGTGTCCATAAGTCCATTTCCCTTTTTAAACTCCTCAAAC
CATTTGTTCATCTCATCTTTGCTGTTCACCTCGATCTTGTTTTCCATTTTGCAGACACTCAAGAGAACACTGTGGGCGTAAGCGGCACGTGTTTCTTTGAGAGGATCCTTCTATAGTGTCACCTAAATGCGCCATATG

>6.AP (Insert sequence below contains BamHI_T7(+)_U04958(-)_NdeI_SP6(-)_XbaI, was cut with BamHI and XbaI, cloned into pET-28c(+) between BglII and XbaI, and finally linearized with SgrAI and NdeI)
GGATCCCGATCCCGCGAAATTAATACGACTCACTATAGGGTCTCAAAGATATCGTTGCCGCACAGCCCTTTATGTGTAAAAACAAAGAAAGAATAAAAGTTGAGTGAAGCAGTATTTGAAGCAAGCATGATTACTAGTTGCAGCCGAAAGCACTGACTTGGAGTTGGGTGGGTGGGATG
CAGAGCACCTGGGCAGCTAGATGATGTTGGCACTGGTGGCATTGCCCTTGACATTGTAGGCATTCTGGAAGGGCGACTGCTTCCCAACCAGGGATTGGTGCAGCAGATGCTCAGAGGCCACGATGTCCATGTCCTTTATGTCAAAGCCCGTCTTCTGAATTTCGAAAAGCTTGACAATG
GTCCTGGCACACGGATTGTTGGACTCAGTGTTTGTCCTTAGATTTAGAATGGACTTGGTGTGTCCAGATCCCTGGGCTGCATCATCTGGGTTGGCTACAGGAATTGTCCCAAAGCAGACACCCATTTCGCTGATCCTACCAGCTGTCAGCACAGCAGGGTGCATGTAGATCCTGTTCTG
CTGGAAAGAGTCATCTGCAAAGAGCTCATAGTGCTTCTTTCCCCACTTCATGGGAGTGCTGAGCAGAGCTTTCTTCATTTTCTTGGTGCCCCTTGGCTGCTTTCCAAGCTCAAAGAGAAACTGGGAAACAGTGGGGAACGTCTCTGGAGTGACCCCTGCCTTGTAGAGCCAGTAGAACG
AGCTGAAAGGAGTGTCAATTTGTGCACCCTGAGCACGAAGTGCAGATGAGTTCTTGTAGAGTTCCTGAGCCTTGGCAATATGTTTCAAAAGGCTTGTCACCATGTTGTCAGCACTTGTCTTGTCGACTTCATCTCTGTGTTTGTCAAGGTAGTCCTTGAGTGCTTCCACAGTCTTTTTG
GCTTCTTCGAAGACTCCTTTACCTTCAGTCTCTGCCAGCTTGGCAAGACCGGTTGCGACAAGTGCAATCCCGGAGCGACCTGATTTGTTGATGTCACCCCAAGGTGGATTGAAGGCCATAATGTACTTTCCCTTGATGAACTCCCTGCACCACTCAATGTGCTCATGACTTACTGGGCC
TCGTGGATTCTCATCGCTGCCTCTGTTAAGGATAATGTTCCTTCTCCTTATCATGTCTGACAGCATCTCTTTGACAGGCACCAAGATCTCTCCTGGAACCTTGTACTCTGCAAGCACTTTGTTGCTCAGCGCTGTTGTGTTAGCATTGATGCGGAAACCTATGTCCTTTCTCCACTTGA
GGGCTGCCTGCTGATAACTAGAGAGCTGCTCTATTTTGGGAATGTCAACCTTCAGCTCAGTGTAGCTCTCGTCCCAGGATTTGATAGTGCTCGTGTTCTTTTCAAACCACTCCAAACCTTTCTTTACAATGCCTGTAGAGCTGACCCAGGCACACTCATATATTGGTGCACAGTACTTG
GTTGCTTCAACAAGAGCAGATGCATATATAGAGTCCTTCTGTGCATCATCGGTTGCACTGGCCATCTGATACACAAATCTGTCCAAGTTCGGCACATTTTCACAAAAAGAATATGAGTTTGTGAAGGTGTCCACAAGCCCATTTCCTTTCTTGAACTCCTCAAACCACTTGTTCAGATC
GTCTTTGCTGTTCACCTCGATTTTGTTCTCCATTTTGCTTACACTCAAGATAACACTGTGGGCGTGAGCGGCACGTGTTTCTTTGAGACATATGTTCTATAGTGTCACCTAAATGCGCTCTAGA

Figure A. DNA templates for in vitro transcription of the S segment from CCHFVs. DNA insert sequence, presented in the FASTA format, consists of the indicated restriction sites (underlined), T7 promoter (in green) with positive polarity (+), CCHFV sequence with negative polarity (-), which is opposite to that based on the GenBank accession number, as well as SP6 promoter (in blue) with negative polarity (-). The insert was chemically synthesized and cloned into the pET-28c(+) plasmid vector based on restriction enzyme digestions and ligations as described. Note that BamHI and BglII-digested products have compatible cohesive ends that can be ligated during cloning. SgrAI is a nearby restriction site upstream of BglII in the vector. The resulting plasmid was linearized by restriction digestions and then served as templates for T7-based in vitro transcription of a negative-strand RNA corresponding to the S segment of the CCHFV genome. The introduction of the SP6 promoter was intended to facilitate SP6-based in vitro transcription of a positive-strand RNA in case needed. In this study, only the negative-strand RNA was used.

>Hazara (Insert sequence contains BglII,T7(+),NC_038711(-),BamHI,SP6(-)and XbaI, was cloned into pET-28c(+) between BglII and XbaI, and finally linearized with BglII and BamHI)
AGATCTCGATCCCGCGAAATTAATACGACTCACTATAGGGtctcaaagatatcgttgccgcacagccccaaattttacaaaacagaatagaataagaatgaaagtaagaatagaagcaaagcaatgcagaagaaaaacagcagtagagatgtgttggcagtgaacctagccgcaatcct
agatgatgttgatgttggtggcattgcctttaatgaggtaggcgttttggaagggcgagcgcttccccacaagagattgatggagaagatgttcagaggcgactatatccatgctctcaatgtcataacctgctttttggatctcaaagaggcttgaaatgatgcaggcacacgggttg
ccgacctcggtcttggtcttatagttgaggactgcctttgtgtgcccggacccttgggcggcatcatcgggactggtgacgggcacggctccaaacgatattccaagctcggacattcttccagatgttagcacacaggggtgcatgtagattctgttctctgtgaagtcgttatcagc
aaacagttcaatgagccttttccccatttgagaggggtgttgatgagtgccttttgcatttttttctgtcccttggggttcttacccagctcgaaaaggaattgggacacagatgggaaggtgactggtgtcactcctgccttccaaagccagtagtagctgctgaacacagtgtcta
tttgagctccctgggcacggaaggcgttcgactgctttgccagagccagggcagtcttgtagctttctctcacccctttaggaggtcttctgccttgtcttggtcgacttggtctttgttctctttgacccatccttcaagttgtgcaatgcttgccttggccttgtccatcacgtct
ttcccctctagctcaaccagctttgctaatcccgtggccaagagagggtacccagatttacctgccttgttaatctctccccaaggagggttgaaagcattcaaaaatttgcctgaggcaaactctctgcaccagtcaagatgctcacgcccaacaggccctctctttggcgcatcatc
ggagccaccgttgatgatcctgttcctgcgtcgaatcatatcgctgagcatttctttgatgttattgatcactgtccctggcaccttatactctgccgccactgcctttttttagtatactggtgtactccccaatggagaagccgacatcctttctccagttgagggcggctttctggt
atgccagtaatgcctcagcaggcggtgtctctgttcttagcttttggtagttggcatcccagaccttcacggtgtcagagtctttgtttttgtcgaaccattccagacctcttttaactgtcccagtgcaggaagcccatgcacactccatgataggagcacagaatcgggtggcctca
atgagtgctgagctgtagatggcgtccctctcattgtccgtgctggctagctccatcttgtacttgtaggtgtcgagctgaggaatttcggcacaaaaggatgcagattccgtgtacttgttgttcagtttgtgtttctcagcaaactgcttgtaccaggtgttgaactcttccttagt
actggcaacaatcttgttctccattctcgctgatgttgtgaggcgaccggttttgctctcaaaaggatgaaaacgtggggcgtctgcggcacgtttgtctttgagaGGATCCTTCTATAGTGTCACCTAAATGCGCTCTAGA

>NSD (Insert sequence contains BamHI,T7(+),AF504294(-),NdeI,SP6(-)and XbaI, was cut with BamHI and XbaI, cloned into pET-28c(+) between BglII and XbaI, and finally linearized with SgrAI and NdeI)
GGATCCCGATCCCGCGAAATTAATACGACTCACTATAGGGtctcaaagagatcgttgccgcacagccccttgttaataaaagcagaaataaaagaaacagaaagaagcaagcagcaggtagcggttcggctagatgatttggatactggtggcattccctcggatgttgtaggcattc
tgaaaaggggacctctttccaacaagtgactgatgcagcagatgctctgaggcaactatgtccatgctctcaatgtcgaagcctgctttttgaatgttgaacaaggcaacgatgttctgggcacaggggttaccagcctgggtgtcagtcttgtagttcagtatgttcttgatatgacc
tgaaccctcagctgcatcatcaggattggccactggaattgccccaaagcaaacgccaagctccgacattcttcccgctgtcaagactccagggtgcatgtaaatccgattggcagtgaagctatcatcagcaaagagtgcaagtaatttctttccccatttcagaggcatgcttgaca
gtgctttcttcatttttttgttccctctgggcaccttgcctagctcaaagagaaactgtgagactgtcgggaacatctctgcagtcacacctgccttccaaagccagtagtagctgctaaaaacagtgtcaatctgggctccttgagctctcatggcacttgactgcttggctagttca
agggctttctgcaggtgcttggtcaaaccgtcaacaacctctttgccattctccacttcatccttgtgggcattgatccacgccacgaggcttttcagagactctttcaagtcctctgcaactttgggaccgtccagctctatcaattttgccatgccggttgctgctagagcaattcc
tgacttccctgccttgttgatatcaccccatggtgggttgaaaacaacaaggaacttgccctgtgcaagatctctggcccagtcaatgtgctctttgctgacaggtcccctctttggaacgtcttcactgccgtctctgtttaggattttgttccttctcctaatcatgtcggatagca
tgtccttgacgtttactacaattgaaccagggactctgtactcggttgcaattgcagcagccaatgccgctgtgtttttgttgatcccatatttggtgtcctttctccactttagagcagccctctggtaacctagtaactgttcaggactgggtacctccgttctcagtttcccataa
tctgcatcccagatcttcaccatctctccgttgttatcaaaccagtcgaggccacgttggatcatccctgtgcagctggtccaggcgcactctaagataggagcacagaacttagttgcagcaaccaaggcagaagcatatactgaatctttctcactttcagatgaaacaagcctcat
gctaacctcatatcctgacaaatcaggtatggtttcacagaaagaagctgaattagtcaaaacagacttaagtttgtgcttctcgctgtaggttttgtgccatgcaaggatggcatccttgttgtctgcgacaatctggttctgcatggtcaaagttcaaagacaattcgggcgaaagc
ggcacgtgtgtctttgagaCATATGTTCTATAGTGTCACCTAAATGCGCTCTAGA

>Dugbe (Insert sequence contains BamHI,T7(+),AF434164(-),NdeI,SP6(-)and EcoRI, was cut with BamHI and EcoRI, cloned into pET-28c(+) between BglII and EcoRI, and finally linearized with SgrAI and NdeI)
GGATCCCGATCCCGCGAAATTAATACGACTCACTATAGGGtctcaaagagatcgttgccgcgcagcccccctgtgttaagcagaattagcagattgattacgacttacactatgttagtatgttagtcaaaaaagcaaaactaatatataaagaaggaaaaatggaaagataagcaaga
aaaaagcttttagtggcctttcgctgatgtgcaacttgtttaagatgttagggaaggccacacaatgccagcttttagatgatgttaacattggttgcatttcctctcaccttgtaagcattctgggttgggcatcttttaccaacaaatgactggtgcaaaagatgttcagtagacac
tacttctaagctttccagatcaaagccagatttctggattctgtatagttgcacgattgtggatgcacatgggttctgcacactggtgtcaattttgaagttcagtatgttttttgagtgtcctgaacctaggattgcatcttctgggcttgcaacaggaataaccccaaagcatgcac
ccatttcactcattcgaccaggagtcagaactgcaggatgcatgtatagtctgttgcccaaaaagtcatcatctgcaaacaggttaatcaatcccttgccccaccttagtggtgtggaagtcagtgctttgatcatttttttgcctcctctagcactctggcccatctcaaagaggaat
tgagacaaagtgaaaaatgtctctggcttaacaccagcgctccaagcccaataataggctgagaaaggagtgtctatttgtgctccttgtgcacgcagggcattggacttttggatagttccactgcttgagccagatgctttgtcatagtctggacgagctcattgcctttcccatc
ctctagtgcatccttgttatcttcaacccatttctttagatctagaagggcctgccttatatcttcaagcttctttgacccatctagctcagcaaccttcaccatggcagtagctgctagaggaatgcctgatttaccacacttgtttatatccccccaaggaggattaaacacaactt
ggaactttcctcctgccaactcacggccccaagagacatgctctctgctgataggaccttttttcccagcatttctccgtctccgttcagcaggacattcctgcgcctgatcatgtcagacagcatttctttgacatcaacagcaatttctccaggcactttgtactctgcaacaact
ggatgtgtgagagaccttgtgaattggttaatttcataaccaacgtcctttctccacttttgggccgcctgctggtatgaaactagttgctctggttcagggataccctttctgagatccatgtactttgcgtcccagagcttgatgaagtctttgttcttatcaaaccagcttaagct
cttctgcactacgcctgtactgcaggtccaagcacactcataaattggagcacagaaacgtgttgcctccaccagagcattagaaaagattgagtctttttctttatcatctgtggcaagggccattttcatttctgctagagctaggtcaggtactctatcacacagggatgcagagt
ttgtgaggtttgaccttaattggagcttttcactaaaaggtttaaaccactcatcaaactctttttttattgtttgccttgatctgattctccatcttgcttcttcaaagtacaatcgggcgactgcggcacgtttgtctttgagaCATATGTTCTATAGTGTCACCTAAATGCGCGAAT
TC

>Oropouche (Insert sequence contains BglII,T7(+),KP052852(-),BamHI,SP6(-)and XbaI, was cloned into pET-28c(+) between BglII and XbaI, and finally linearized with BglII and BamHI)
AGATCTCGATCCCGCGAAATTAATACGACTCACTATAGGGagtagtgtgctcccaattcaaaaatacgtttctaaaatgacatagctttccccaaccacccaacccttttataatattacagctgcagttctttttttgtgctttcgcactttttttatttattttatatatttttttaa
tttttttgtattttgttatttatttctttttttgttgtttttttgtgttttatattttatttgtttactgtactccactatatgtcaattccgaattggcgcaagaagtctcttgctgctggggagaatccagttctggcccaggccagagtgcctacagcttcaaaagctgctctt
acagcaactatttcttcacgcatccattgctcggcagtcttttggccataccgttgccggaggactttcttcataaagttgacatccatgccatccttgagaaccctatgcatctcaatcaccaaagggaagaaagtgaatgtttgcaggaacatctcagcaccagcagcaaaccccag
gtacattgcgtcaccatcattccaagtacagccctttacctcagccaaaggcactactacagtggctctgaactcagcttgcttaattggtgatcgcatctgggtaaaggcccagcgcgctaggtatcctgacagacggtgcagggtaagaccgttgtccggcactggattcgactgga
actgtgggaaatggttattaaccattgcaaattggaccccaccaaaagtaaggtcaaccatgggcctcgatgtcttacgtaagacatctttggccttcttttggttgaggaagaagactctaacaacaccagcattgagcacttgtccgtatctagcttcaaatgccacatatgctgcc
tccggatcaaatgtagatgtagtccgttgtggtacatcgttgaaaatgaactctgacattattggaatttctttttatgttttgaattgtggagtacactactGGATCCTTCTATAGTGTCACCTAAATGCGCTCTAGA

>Hantaan (Insert sequence contains BglII,T7(+),M14626(-),NdeI,SP6(-)and XbaI, was cloned into pET-28c(+) between BglII and XbaI, and finally linearized with BglII and NdeI)
AGATCTCGATCCCGCGAAATTAATACGACTCACTATAGGGtagtagtatgctccctaaaaagacaatcaaggagcaatcaaatgaggtagttaagttgaggtagttttgtgttgaggtagaatgttgttgttagtaagcagagaaagcagaaagaaagggaaagaaaacaagattagtt
aaggatcaaatgaaatctacatccatatatacaaaaaaaggctacttaaggtagcctggagctaaacccacccacaacggattaactgacccacccctgagtaatgattcccacccataaatgttcccatgccctgatatactgattcccctgataataatagtgcaaatatgattgat
aatgattcagtagtatatgataatcacataaaaggattaatacattcattaattagagtttcaaaggctcttggttggagatttccttcactttgacatcaatcaagctctgtgccagtgtccttagctcaggatccatatcatcccctaagtggaagttgtccacagcctcctttccc
caggcaaccatgaagagcacaataattctctggcctagttgtatccccattgattgtgtccttctgaggtaggactgataaaatgatgatttcttccgtagcttctcctcagatgttccaactgtcttagatgccatgattgtatttcgcatgtcctgcaggatggaaaaaaatgcccc
aagctcagcaatacctgctataaacaaacatgttggtggacaacggtctggtgctccagcaaaaacccatattgatgatggtgactcaatatcttcaatcatgctacagccagctgcttctgcatgctggcgtatagcctttgactcctttgtctccatattgcctaatgccacttgcc
gctgccgtaagtagtccctgtttgttgcaggaccaccaaggaggctaactgctgctgtatctggaagaagcttgcaaggttcaattaaccattgttcgatacgatcactccagtcctttgctaatgctagaaaaccaattacactcataactggactgatcatctgccgtgccttaatc
tgtgcagggtagagcccacagactgctgttctatatctaccaggtgtaatctcttctgccttcatgcttgactgtgcatttggcaaggacacgtaaagatgttttggtttccggataccgttaacatcctcgaacgagctatcatccttaaatcgaatccgggtccctttattatcctt
ggtagtttgcctccccttgttgtcaacatatacagagctttcagaagtatcgggacgacaaaggatgtaagatagacgatgatgctcagccagtctgctgtctgtcctgtaggttcatcaatatccaaatggtttaaatccagcacattaccataactgagcattgacctctctttca
gatggtctccaggctccacccctgttggatcttgttccttcccaaggtttttcccagttgcaatcctatctgccagttgcctttttaactcatcaattttgcctggatagatactgcaacgccctctcggtcagttaatgttctcttgttcaactcatctggatccttttcatactgt
ttttctgcatccctcaccttctgcctggctatcactaattgaccctcatgggcattgatttccctctgtaattcctccatagttgccatcgttgttctagtagctctttagggagtctactactaCATATGTTCTATAGTGTCACCTAAATGCGCTCTAGA

Figure B. DNA templates for in vitro transcription of the S segment from CCHFV-related viruses. DNA insert sequence, presented in the FASTA format, consists of the indicated restriction sites (underlined), T7 promoter (in green) with positive polarity (+), viral sequence with negative polarity (-), which is opposite to that based on the GenBank accession number, as well as SP6 promoter (in blue) with negative polarity (-). The insert was chemically synthesized and cloned into the pET-28c(+) plasmid vector based on restriction enzyme digestions and ligations as described. Note that BamHI and BglII-digested products have compatible cohesive ends that can be ligated during cloning. SgrAI is a nearby restriction site upstream of BglII in the vector. The resulting plasmid was linearized by restriction digestions and then served as templates for T7-based in vitro transcription of a negative-strand RNA corresponding to the S segment of the viral genome. The introduction of the SP6 promoter was intended to facilitate SP6-based in vitro transcription of a positive-strand RNA in case needed. In this study, only the negative-strand RNA was used.

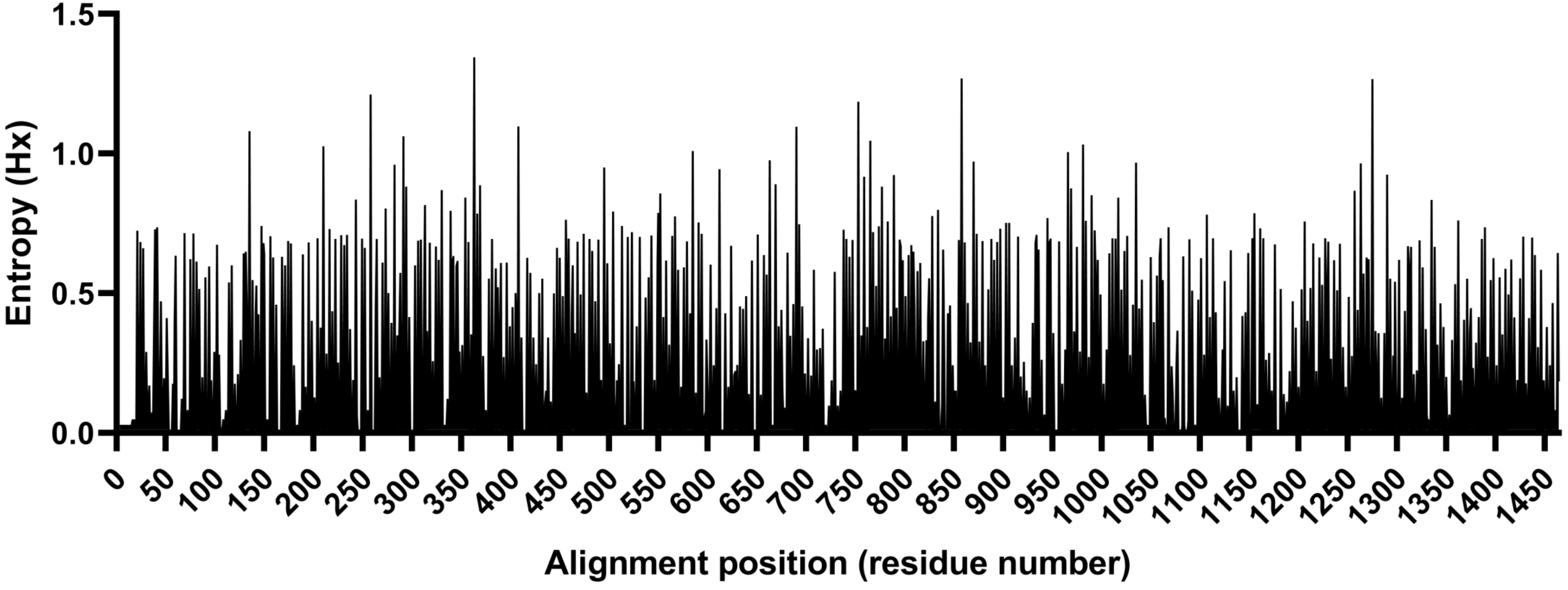

Figure C. Shannon entropy plot of 265 CCHFV isolates. This involved all the S segments that we found with a complete sequence described in GenBank. The S segment sequences were aligned using Clustal W (v1.6) in MEGA 7 (v7.0.26) [1]. Shannon entropy analysis was conferred using BioEdit (v7.0.5.3) and plotted on GraphPad Prism 9 (v9.0.2). Residues with entropy values higher than 0.2 are considered variable and below 0.2 are considered conserved. There is a noticeable variability plateau amongst most positions at an entropy value of 0.75. Hyper variability is observed at 54 positions, with entropies above 0.75.

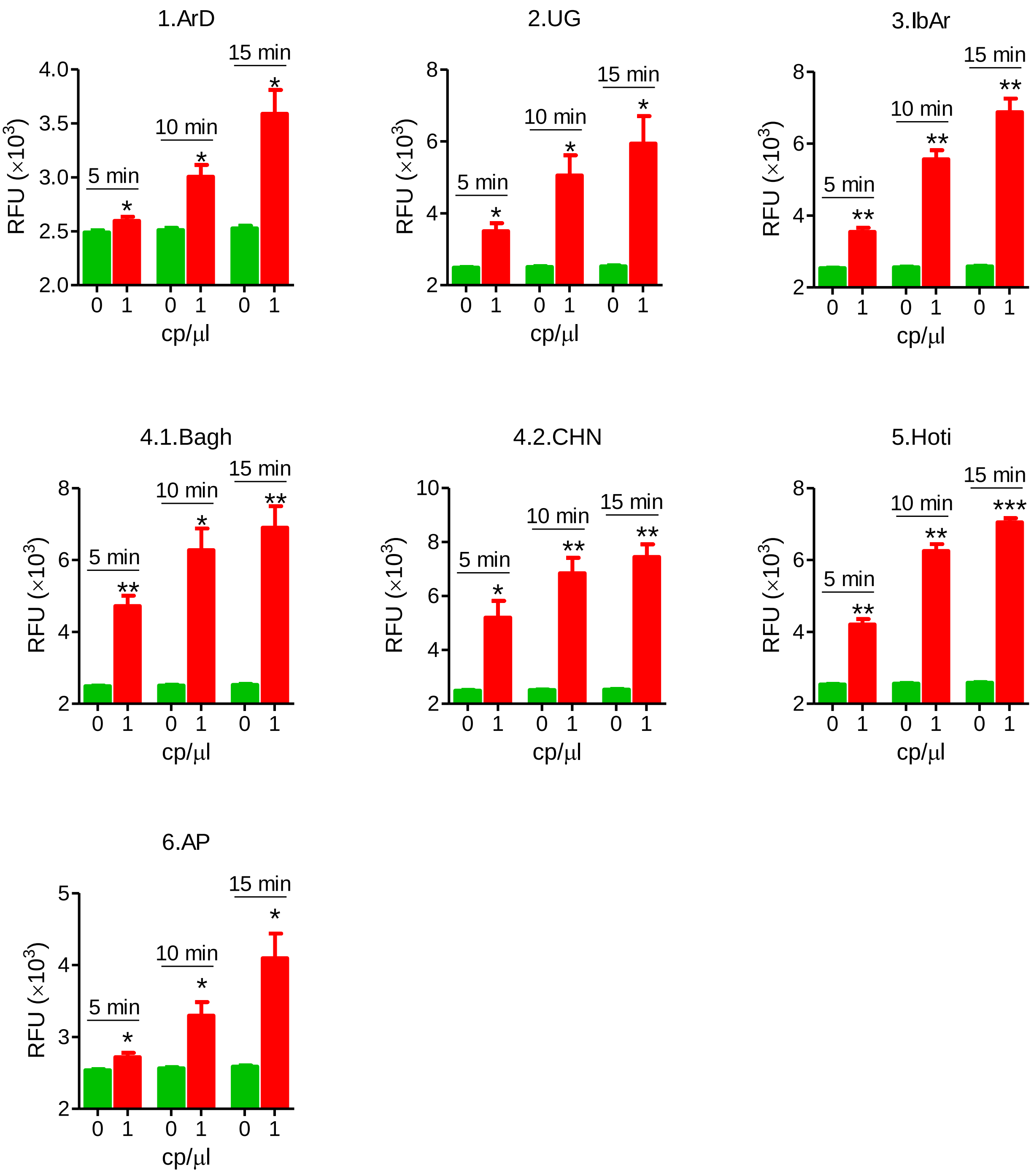

Figure D. The degenerate sequence-based CRISPR diagnostic generates significant amplification signals rapidly at 1 cp/µl of CCHFV RNA from any clade/sub-clade. Data from Fig 3A-G were statistically analyzed, comparing the amplification signals between the conditions with 1 cp/µl and 0 cp/µl (NTC) CCHFV RNA in the T7-Cas13a reaction, at early time points of the reaction (5, 10 and 15 minutes). Graphs are based on three independent experiments. Significant differences in RFUs were determined by paired *t* test: * $p < 0.05$, ** $p < 0.01$, and *** $p < 0.001$.

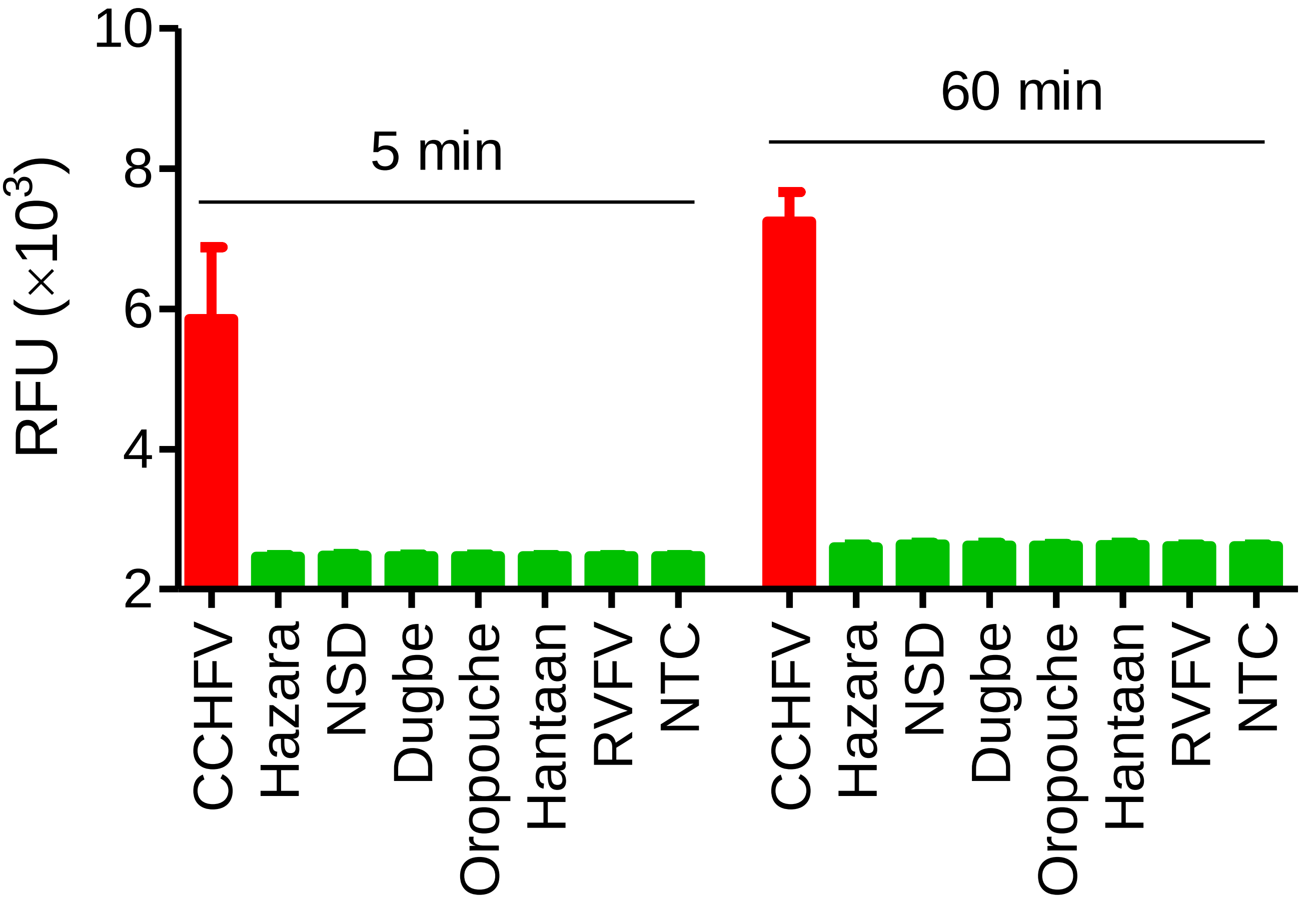

Figure E. Significant signal amplification with CCHFV but not closely related viruses. Data from Fig 3I were statistically analyzed, comparing the amplification signals among different viruses, at an early or late time point of the T7-Cas13a reaction (5 or 60 minutes). Graphs are based on three independent experiments. Significant difference in RFU was found by paired *t* test ($p < 0.001$) between CCHFV and any of the other viruses at either time point.

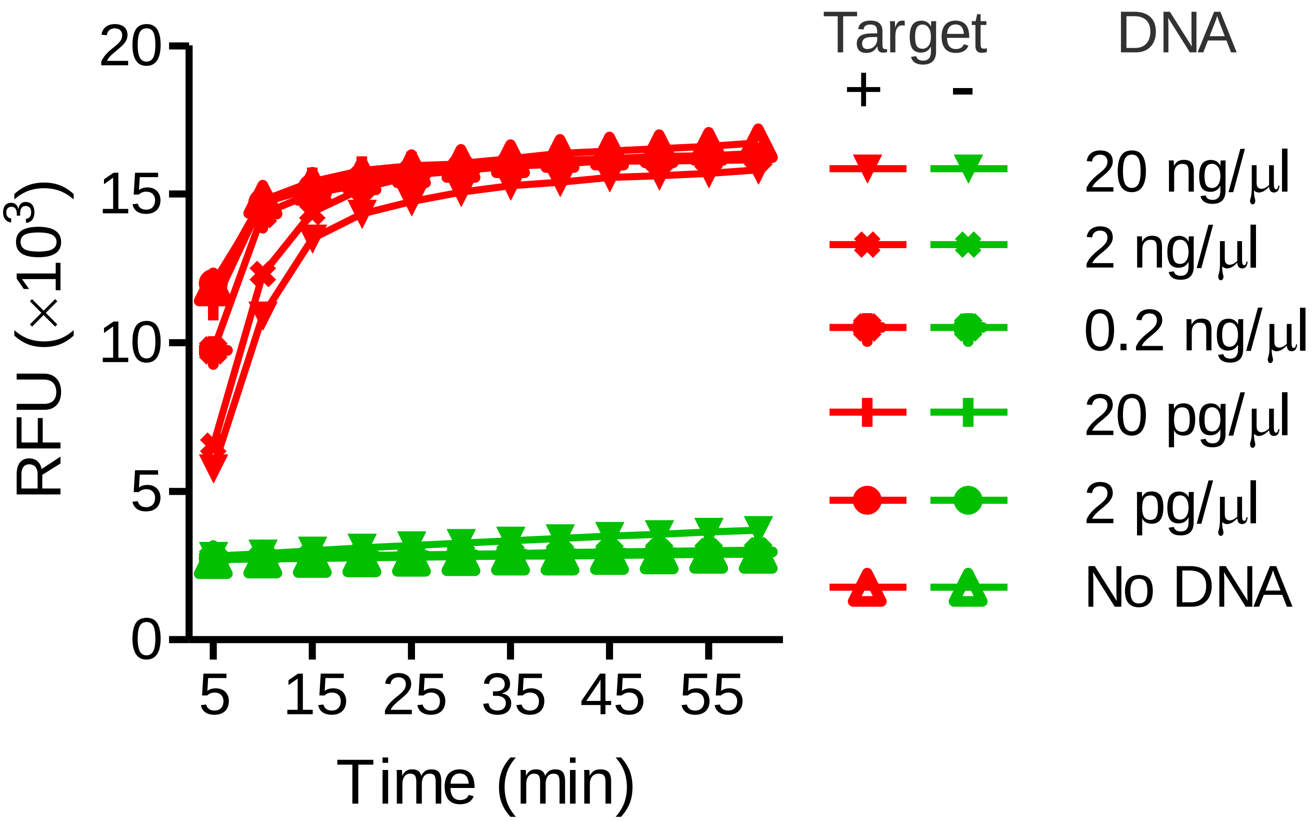

Figure F. Human genomic DNA contamination does not lead to false positive signals in the degenerate sequence-based CRISPR diagnostic for CCHFV. The assay was tested in the presence (+) or absence (-) of target CCHFV RNA (Hoti strain), and with or without added human genomic DNA (Sigma,11691112001) at an indicated concentration. Amplification plot shows relative fluorescent units (RFU) at indicated time points. Data are represented as mean of three technical replicates.

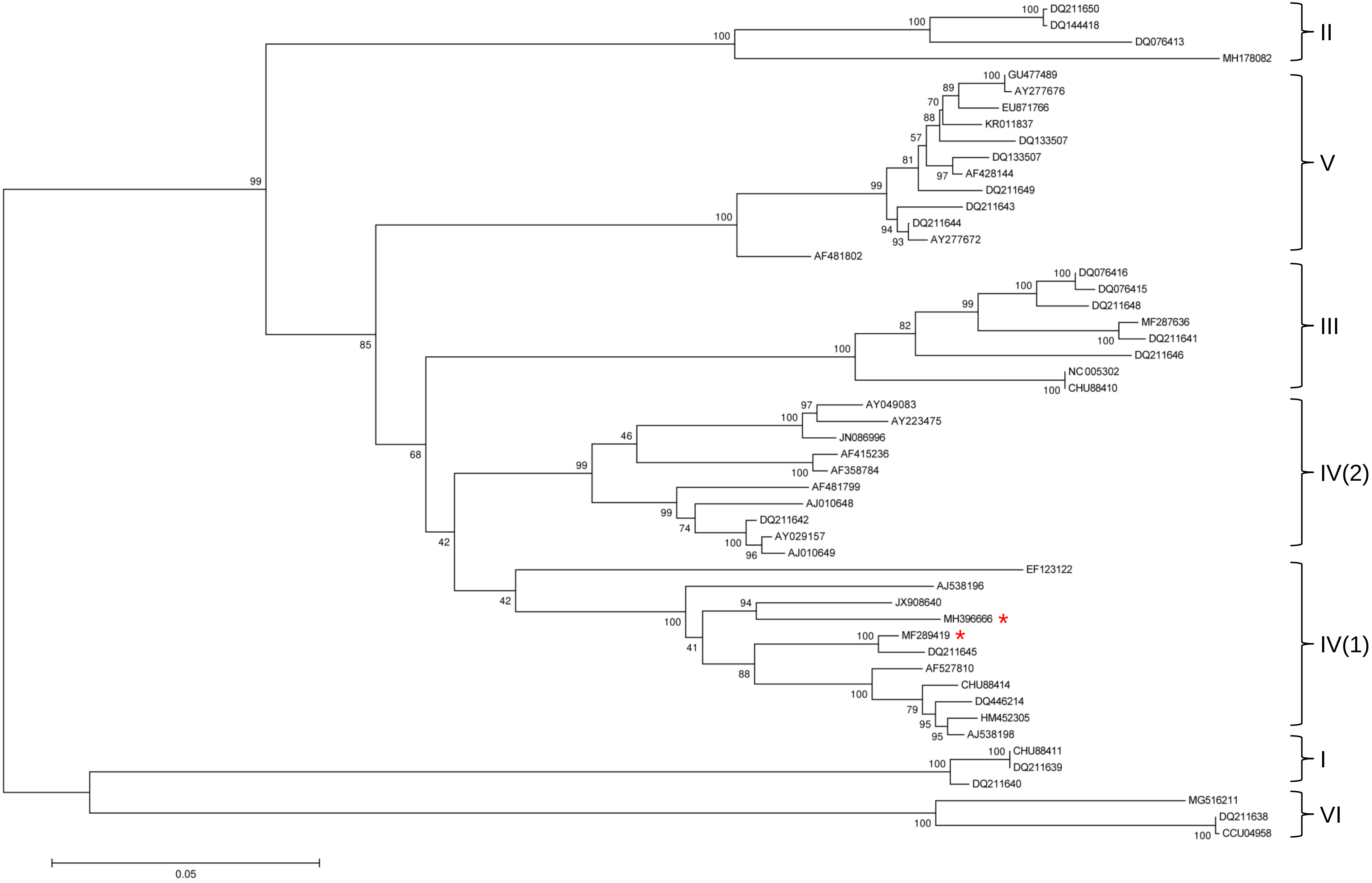

Figure G. Clade location of the IND and UAE isolates. Two recent CCHFV isolates from India (IND, GenBank accession number MH396666) and United Arab Emirates (UAE, GenBank accession number MF289419), respectively, were analyzed for phylogenetic relations to 49 traditional CCHFV strains with known clade distributions, based on the S segment sequences. Phylogenetic analysis of these was performed by using the Maximum Likelihood method based on the General Time Reversible Model with Invariant sites using 1000 bootstrap replicates [2]. Branch lengths are measured using number of substitution per site. Phylogenetic tree is labelled with clade numbers. The IND and UAE isolates are each indicated by a red star. They were both located in Clade IV(1).

References

1. Kumar S, Stecher G, Tamura K. MEGA7: Molecular Evolutionary Genetics Analysis Version 7.0 for Bigger Datasets. Mol Biol Evol. 2016 Jul;33(7):1870-4.

2. Nei M, Kumar S. Molecular Evolution and Phylogenetics. New York: Oxford University Press; 2000.
