# Supplementary material for: Degenerate sequence-based CRISPR diagnostic for Crimean–Congo hemorrhagic fever virus
Source: PLoS Negl Trop Dis. 2022 Mar 10;16(3):e0010285. doi: 10.1371/journal.pntd.0010285 (PMC8939784; doi:10.1371/journal.pntd.0010285)
Supplement: Attachment — Submitted filename: Response to Reviewers.docx [file pntd.0010285.s004.docx]

**Point-by-point responses to the Reviewers' comments**

**Reviewer # 1**

**Methods**

Reviewer did not comment.

**Results**

Reviewer did not comment.

**Conclusions**

Reviewer did not comment.

**Editorial and Data Presentation Modifications?**

Reviewer did not comment.

**Summary and General Comments**

*In this study, Hongzhao et al. proposed and aimed to test a design of CRISPR-based in vitro diagnostic assay based on degenerate gRNA. Even though it's an interesting idea, some major pieces of information were missing in the study which significantly compromised the quality and conclusions of the study.*

***1)*** *The authors claimed that CRISPR-based assays were highly intolerant to mismatches, and therefore a degenerate gRNA would be needed for viral detection. However, no experiments at all were performed to validate such a presumption, other than a few citations. Speaking from our own experience, there was a decent degree of mutation tolerance in these assays which allowed detection of pathogens even in the presence of natural variations. This presumptive hypothesis, which constituted the foundation of the current study, has to be carefully tested before introducing degenerate designs.*

**Our response:** We thank the Reviewer for the extremely helpful insights. Accordingly, we have revisited the presumptive hypothesis, which was built upon implications from previous studies, by performing new experiments. Our new data are consistent with the experience shared by the Reviewer in that “there was a decent degree of mutation tolerance in these assays”. Therefore, in the revised manuscript we have deleted the overstatement of extreme mismatch intolerance by CRISPR, and have described the rationale and significance of the degenerate sequence-based CRISPR assay more carefully. Overall, our evaluations demonstrated that the degenerate sequence-based CRISPR diagnostic for CCHFV is functional and has maintained the advantages of traditional CRISPR diagnostics (isothermal, sensitive, specific and rapid). Furthermore, with new findings and considerations, the study has also suggested that the degenerate sequence design brings additional advantages, including a built-in confidence in mutation coverage, an enhanced capacity of mutation tolerance and the flexibility to be quickly adapted to emerging mutations. Therefore, the degenerate sequence-based CRISPR diagnostic holds great potential to serve as a functional alternative to traditional assays and a useful tool for detecting pathogens with variable regions. These comments have been systematically elaborated in the Discussion section (Page 25, Lines 467-590) of the revised manuscript, while related data and descriptions distributed in locations of the other sections of the manuscript (which are too many to list one by one here).

***2)*** *There appeared to be a logic paradox between the mismatch-intolerant theory and the assay design. If a single mismatch between the gRNA and the target would abrogate the binding and hence the detection, the concentration in a degenerate gRNA design would need to be much higher than a single-sequence one in order to reach the same effective concentration. Say only to cover 4 degenerate NTs, a degenerate gRNA pool would consist 256 (4x4x4x4) difference sequences and only the one that match exactly to the target would work, which made the effective concentration as low as 1/256. In this study, the authors did not provide evidence proving that the degenerate design indeed contribute to the detection.*

**Our response:** We thank the Reviewer for this in-depth reasoning. As discussed above, our new data are consistent with the Reviewer’s experience, supporting a decent mismatch tolerance. We have made corrections and updates accordingly, as indicated in the above response to Comment # 1).

***3)*** *The design of analytical assessments of an diagnostic assay needs to be improved. For instance, assay's LoD should not be evaluated based on the statistical difference between positive and negative samples. Rather, an assay cutoff should first be established and then technical repeats should be performed to determine the concentration at which at least 90% of the repeats were positive.*

**Our response:** We thank the Reviewer for suggesting a more standard way of assessing the LoD. Accordingly, in the revised manuscript, an assay cutoff of negative threshold was established as mean + 3× standard deviation of no-template controls (NTCs). A relative fluorescence unit (RFU) value above the negative threshold is considered CCHFV positive. The LoD was defined as the lowest concentration at which 100% of the repeats were positive (Methods section, Page 15, Lines 262-266). Based on that, the assay was found to have an LoD of 1 cp/µl (Fig 3H, and Results section, Page 21, Lines 386-391).

***4)*** *No clinical specimens were tested at all. To develop a diagnostic assay, the sample type it could be used in should be defined and validated, due to the fact assay performance could vary greatly in various samples. Also, interference of human DNAs is another factor which should be considered and tested in both analytical, mock settings as well as clinical samples.*

**Our response:** We do not have access to CCHF patient samples for the time being. We agree with the Reviewer that contaminants potentially carried from clinical samples could affect the assay performance. A major one of these, human genomic DNA, is commonly known to interfere with the specificity of nucleic acid tests, causing false positive signals. By spiking human genomic DNA into CCHFV RNA samples, we validated that it does not impact the specificity of the CCHFV CRISPR assay under the conditions tested (Fig F in S1 File, and Results section, Page 22, Lines 410-419). In addition, since a degenerate sequence has never been used in CRISPR, the study has primarily been designed, on a proof-of-principle basis, to focus on an initial test of the feasibility to use a degenerate sequence-based CRISPR against highly variable targets. For that purpose, the assay has been tested using multiple types of CCHFV RNA sources, including chemical synthesis, enzyme-based *in vitro* transcription and cell culture propagated, whole viruses. We believe these findings, based on the above listed sample types, have fulfilled that study scope. These and additional, related discussions and perspectives have been included in the revised manuscript (Discussion section, Page 28, Lines 547-580).

**Reviewer # 2**

**Methods**

*The use of degenerate sequences for successful CRIPR-Cas13a detection of genomic variants of Crimean Congo Hemorrhagic Fever virus CCHFV is presented by Li H et al. The results were obtained using synthetic RNA targets.*

*Only one genome type of one representative of CCHFV was utilized for confirmation of the technique. The paper shows a potential technique to detect CCHFV which will include genomic variations previously reported.*

*Degenerate sequence-based CRISPR diagnostic to cover viral diversity as a title would be too general, as the paper describes a specific example of diversity in the genomes of CCHFV.*

*The proposed objective to apply degenerate spacer region for the detection of other viral genomes was not provided, data regarding CCHFV shows a successful approach. The title may reflect the work specifically done for CCHFV detection.*

**Our response:** We thank the Reviewer for these important comments and suggestions.

Regarding genome types of CCHFV representatives, the degenerate crRNA sequence was designed using traditional CCHFV strains (Fig 2B). As the Reviewer suggested, the design is expected to cover genomic variations previously reported but a possibility needs to be considered that new mutations could develop in newer and more divergent viral isolates. We have conducted an extensive BLAST search and confirmed that all CCHFV variants except a few are fully covered by the degenerate crRNA sequence, in terms of both the positions and nucleotide types of mutations. Through additional experiments, we have confirmed that the degenerate sequence-based CRISPR assay can successfully detect those exceptional mutant sequences (Fig 4). The data demonstrate that the assay is capable of detecting emerging viral variants with new and more complex mutations. These findings are described in detail in the Results section (Page 22, Lines 421-461).

Concerning the title, we have made it CCHFV-specific: “Degenerate sequence-based CRISPR diagnostic for Crimean–Congo hemorrhagic fever virus”.

**Results**

*The results could be summarized in two to three tables and figures, tables provided can be combined. Results can be compressed. The off-target detection of related viruses did not include other closer viral genomes, instead included very distantly (phylogenetically) viruses to prove specificity of the method. There is no nucleotide sequence homology among those viruses.*

*Figure 2 lists several gene bank accession numbers for the S region, but U04958.1 corresponds to the N region. Please clarify which accession numbers were for S and include most other distantly related CCHFV S sequences in the analysis.*

**Our response:** We thank the Reviewer for the very helpful suggestion and reminders.

We have compressed data presentations accordingly. The previous Tables 1 and 2 have now been replaced by texts in the updated Methods section (Page 9, Lines 179-188). The previous Figs 2 and 3 have been combined into the new Fig 2. The previous Fig 5 has been moved to supplementary, and is now Fig D in S1 File. The previous Fig 6A has been combined with previous Fig 4 as part of a new Fig 3, and previous Fig 6B has been moved to supplementary, now new Fig E in S1 File. In addition, the revision has brought a new Fig 4. Altogether, these rearrangements have led to a reduction from five tables and six figures down to three tables and four figures, which include new data.

Regarding CCHFV-related viruses for specificity testing, we included popular members from the same family as CCHFV (Bunyaviridae). These viruses have been used in negative viral panel testing to validate the specificity of a commonly used CCHFV Real-Time RT-PCR assay (Atkinson 2012, PMID 22217175). Among them, Hazara, NSD and Dugbe viruses are species within the same genus as CCHFV (Nairovirus). Moreover, while species in the Nairovirus genus can be further divided into a few serogroups, Hazara and CCHFV belong to the same serogroup (CCHF group). Thus, these species are considered good representatives of close CCHFV relatives. We agree with the Reviewer that the remaining CCHFV-related viruses used in our negative panel testing, belonging to other genera, are not the most closely related to CCHFV. The rationale was that we would like to expand the test scope to different genera in the same family, instead of limiting it to a single genus. Indeed, the Atkinson 2012 study also included viruses from different genera of other families. Therefore, we hope that our specificity test has included viruses that are among the closest relatives of CCHFV and has covered a reasonable range of targets.

Concerning the genomic region represented by GenBank accession number U04958.1 in Fig 2, the definitions of the S region and the N region are largely interchangeable. The CCHFV genome consists of three viral RNA segments. One of these is the small (S) segment (or the S region/gene). The S segment encodes the nucleoprotein (N protein), and is thus alternatively called the Nucleoprotein (N) gene (N region). Therefore, the description title of U04958.1 in GenBank as “Nucleoprotein (N) gene”, also means the S segment. We thank the Reviewer for reviewing the details carefully and noting the discrepancy. We have added the clarification in the revised manuscript (Page 8, Lines 158-161).

Regarding the inclusion of other most distantly related CCHFV S sequences in the analysis, we have added the revision work accordingly. Our new data demonstrate that the assay is capable of detecting emerging viral variants with new and more complex mutations. This has been discussed above, in our response to the Reviewer’s comments on Methods. The new, more distantly related viral variants that we have tested include MH396666.1 from India, the one also suggested by the Reviewer in their comments on Conclusions (see below).

**Conclusions**

*The main limitation found was that there are a large number of virus isolates with as low as 86% nucleotide identity (e.g. MH396666.1 from India) that should have been included in the BLAST comparison of Figure 2? what is the degree of nucleotide variation found amount all known CCHFV S gene regions?*

**Our response:** We greatly appreciate the insightful comments and especially the recommendation of MH396666.1 from India. This is a major CCHFV strain we have used accordingly, to validate that the degenerate sequence-based CRISPR assay is capable of detecting emerging viral variants with new and more complex mutations. We have performed a BLAST search and comparison including this strain and have confirmed its detection by the CRISPR assay (Results section, Page 22, Lines 421-443, Fig G in S1 File and Fig 4A).

The degree of nucleotide variation among CCHFV S gene regions was previously reported to be 20%. A similar number, 20.152%, was found in our confirmatory phylogenetic analysis (S2 File) and multiple alignment analysis (S3 File), based on all CCHFV isolates that we found with a complete S segment sequence described in GenBank. We have added a new paragraph in the Results section describing updates on sequence divergence in CCHFV (Page 17, Lines 290-299). In addition, we would like to include a new coauthor, Kielich D, who has performed the related revision work, which also includes Figs C and G in S1 File.

**Editorial and Data Presentation Modifications?**

*Minor modifications include correction of few typos (line 310), and simplifying the sentences throughout the results and discussion sections. Also, Abstract narrative should not be repeated in the Introduction section.*

**Our response:** We have made modifications accordingly, including correction of typos and simplification of sentences throughout the manuscript. In the updated Abstract, we have also tried to avoid its narrative being repeated in the Introduction section.

**Summary and General Comments**

*Overall, the paper proposes a potentially good molecular method to detect CCHFV. The paper may benefit from including more detailed comparisons of existing genomic variances. The list provided intends to include the most recent and diverse variants?*

**Our response:** This has mostly been discussed above, in our response to the Reviewer’s comments on Methods. The degenerate crRNA sequence was designed using traditional CCHFV strains (Fig 2B). To confirm its coverage of existing genomic variances and the most recent and diverse variants, we have conducted an extensive BLAST search and found that all CCHFV variants except a few are fully covered by the degenerate crRNA sequence, in terms of both the positions and nucleotide types of mutations. In new experiments, we have confirmed that the degenerate sequence-based CRISPR assay can successfully detect those exceptional mutant sequences (Fig 4). The data demonstrate that the assay is capable of detecting emerging viral variants with new and more complex mutations. These findings are described in detail in the Results section (Page 22, Lines 421-461).

**Reviewer # 3**

**Methods**

*Methods are clearly explained and well defined.*

**Results**

*Authors have described well and presented. Table 1 and 2 are not needed it should be in text format. Figure 1 should be improved.*

**Our response:** We thank the Reviewer for the very helpful suggestions. Accordingly, these tables have been replaced by texts in the updated Methods section (Page 9, Lines 179-188). We have made clearer labels in Figure 1 and have provided a high quality image file for the figure in the revised manuscript.

**Conclusions**

*How author can reduce time of detection using CRISPR-Cas13 system ?*

*Conclusions and their future perspective should be written clearly.*

**Our response:** We thank the Reviewer for the insightful question and great suggestion. The time of detection using CRISPR-Cas13a system in our current assay was 35 – 40 min (including 25 min RPA and 5 – 15 min CRISPR/Cas13a) to detect 1cp/µl CCHFV RNA. This is typical of traditional CRISPR and shorter than the run time of PCR-based diagnostics, which normally take no less than one hour. For example, a popular commercial kit, RealStar ® CCHFV RT-PCR Kit 1.0 (Altona-Diagnostics), runs about 95 – 100 min (using the supplier’s PCR protocol on an Applied Biosystems™ 7500 Real-Time PCR System). Recent advances in CRISPR diagnostics if adopted in future development are expected to make the assay even more rapid. Some individual assay components can be optimized to enhanced amplification activity of the formulation. The traditional two-step SHERLOCK protocol used here may be combined into a one-pot format, which would allow multiple enzymatic reactions to run simultaneously. This enzymatic multitasking, together with the enhanced amplification activity of the overall formulation, should greatly cut down the run time needed to achieve a clear detection signal. These discussions have been included in the revised manuscript (Page 27, Lines 528-535 and Page 29, Lines 559-574). We have also updated the conclusions and perspectives in an effort to write them clearly (Page 25, Lines 467-590).

**Editorial and Data Presentation Modifications?**

*No comment from the Reviewer.*

**Summary and General Comments**

*I have gone through and found manuscript is interesting for reader and scientific community. Before acceptance for publication, few minor revision needs to be addressed.*

*1. English and typo should be checked in entire ms.*

*2. Abstract should be improved by English for example “The same strategy may also be applicable to other types of CRIRPR diagnostics including those with Cas12a, Cas12b, Cas9 or Cas14…..”*

*3. RPA and CRISPR detection section table 1 and 2 should be written in text donot require in table form.*

*4. How author can reduce time of detection using CRISPR-Cas13 system ?*

*5. Conclusions and their future perspective should be written clearly.*

**Our response:** We thank the Reviewer for listing here these very helpful comments and suggestions. We have checked English and typos throughout the manuscript and made modifications and corrections accordingly. The Abstract has been substantially edited, and the words “The same strategy may also be applicable to other types of CRIRPR diagnostics including those with Cas12a, Cas12b, Cas9 or Cas14…..” have been removed. We have replaced the old Tables 1 and 2 with texts. How to reduce the time of detection has been discussed above, in our response to the Reviewer’s comments on Conclusions. The discussions have also been included in the revised manuscript (Page 27, Lines 528-535 and Page 29, Lines 559-574). We have updated the conclusions and perspectives in an effort to write them clearly (Page 25, Lines 467-590).
