# Supplementary material for: Degenerate sequence-based CRISPR diagnostic for Crimean–Congo hemorrhagic fever virus
Source: PLoS Negl Trop Dis. 2022 Mar 10;16(3):e0010285. doi: 10.1371/journal.pntd.0010285 (PMC8939784; doi:10.1371/journal.pntd.0010285)
Supplement: Attachment — Submitted filename: Response to Reviewers.docx [file pntd.0010285.s005.docx]

**Responses to the Reviewers' comments**

**Reviewer # 1**

**Methods**

Reviewer did not comment.

**Results**

Reviewer did not comment.

**Conclusions**

Reviewer did not comment.

**Editorial and Data Presentation Modifications?**

Reviewer did not comment.

**Summary and General Comments**

*As regular, single sequence CRISPR-based assays already showed a sufficient level of mismatch tolerance, the need for using degenerate sequences was not justified. In the revision, the authors acknowledged the above issue, but stated that the degenerate version enhanced the assay. However, lack of analytical and clinical evidences have been showed to support the statement and its significance. In fact, if the assay is highly dependent on the nucleotide specificity of the gRNAs, the degenerate assay would not work due to the very low gRNA concentration of each sequence variant.*

**Our response:** We thank the Reviewer for the critical comment.

We acknowledged in the revision that the traditional single sequence-based CRISPR assays will continue to be a good approach to addressing variable diagnostic targets. The degenerate sequence-based assay was not stated as a required replacement of the traditional assays but rather represents a useful new option in the toolbox of molecular diagnostic methods. However, it does bring additional advantages (as listed below, with enhanced capacity, confidence and flexibility in mutation tolerance against extremely variable targets), while maintaining the major advantages of traditional CRISPR assays (isothermal, sensitive, specific and rapid). These have been supported by the data figures (especially Figs 3 and 4), illustrated in the assay design figure (Fig 2), and have been elaborated extensively in the Results and Discussion sections (particularly Page 27, Lines 514-566).

**The assay has an enhanced capacity in tolerating more complex, higher degree mismatches.** This is clearly evidenced in Figure 4C, where a CCHFV mutant sequence was effectively detected by the degenerate but not the constant sequence-based crRNA (Figure 4C; Page 25, Lines 472-474 and Page 29, Lines 558-560).

**The degenerate sequence-based CRISPR design is also characterized by a built-in confidence in mutation coverage.** While the Reviewer’s and our experiences suggest that “there was a decent degree of mutation tolerance in CRISPR assays”, previous studies did also observe that in some instances, target recognition can fail with as few as two mismatches, and a Zika CRISPR assay was able to distinguish variants differing by one nucleotide. It has not been clearly understood how specific types of mismatches (numbers, locations, nucleotide types involved and contexts of flanking sequences etc.) affect CRISPR detection. Therefore, when designing a CRISPR diagnostic test, an uncertain effect of different types of mismatches should be taken into consideration.

Traditional CRISPR diagnostics, which use crRNAs with a single constant spacer sequence, can be aimed at a conserved region of the viral genome with relatively low extent of variations. The success to cover all (or most) viral variants can be achievable under the condition that a target region is available and identified where all (or most) versions of sequence variations are tolerated by the constant spacer sequence (as experimentally tested, on a case-by-case basis). It is conceivable that the tolerance of some specific types of mismatches between a constant spacer and a mutant target sequence, such as those with number and locations that have not been reported previously (and thus with uncertain effect), may not be confidently predictable during the assay design but can only be determined after extensive experimental testing and optimization. This may often involve the designing and screening of multiple CRISPR sets targeting different genomic regions.

As a new alternative to the traditional constant-spacer CRISPR, the degenerate-spacer CRISPR represents a proof of concept to build “certainty” into the assay design. Since mixed nucleotide types matching all known viral mutations of interest can be directly included in the crRNA design (as exemplified by Fig 2), achieving a coverage of entirety in the very beginning of the assay development process is made possible. This advantage is anticipated to award a simpler and more straight forward path leading to the establishment of a successful CRISPR set that covers all known mutants of a variable viral target (Page 26, Lines 480-512).

**Moreover, the degenerate-spacer CRISPR has the flexibility to be quickly adapted to emerging mutations.** Following the same design principle as in Fig 2, new degenerate nucleotides can be readily added to the crRNA spacer sequence to cover any future CCHFV mutations if needed. This is an extended advantage in assay design, on top of the built-in confidence in covering previously known mutation types (Page 29, Lines 560-564).

Taking these advantages together, the degenerate-spacer CRISPR holds great potential to serve as a new alternative to the traditional CRISPR and a useful tool of choice for detecting highly variable viral pathogens. The above points altogether have been systematically elaborated in the Discussion of the revised manuscript (Page 26, Lines 480-566).

**Reviewer # 4**

**Methods**

*I have no concerns with the Methods section. The Methods are clearly stated in appropriate detail.*

**Results**

*- Please check over the results section on lines 381-391. How was the copy number per ul determined? Based on the sample input volume used in the reaction (line 183), 0.5 ul RNA was used in the reactions, but 1 copy/ul was reproducible detected (line 384). If 0.5 ul were used, the copy number in the reaction would be < 1.*

*- The manuscript would be greatly strengthened if the CCVHF dilutions/LODs were run with a comparator assay (ex. the Altona assay referenced in lines 531-532). Showing a LOD comparison would give the potential enduser a better idea about how your assay compares for more commonly used real-time RT-PCR.*

**Our response:** We thank the Reviewer for the greatly helpful question and suggestion.

**- Definition of copy number per µl (cp/µl) or copy number per reaction (cp/reaction) of the assay.** Since the two-step CRISPR (SHERLOCK) assay consists of an RT-RPA pre-amplification reaction followed by a T7-Cas13a reaction, the definition of cp/µl or cp/reaction was based on the final reaction, the T7-Cas13a reaction.

As follows:

CCHFV target RNA concentration (cp/µl) = Total copy number of target RNA that was input into the final T7-Cas13a reaction divided by volume (µl) of the final T7-Cas13a reaction.

Total copy number of CCHFV target RNA per reaction (cp/reaction) = Total copy number of target RNA that was input into the final T7-Cas13a reaction, which was 25 µl in volume.

We have added the clarification of these definitions in the Materials and Methods section (Page 10, Lines 208-218) and the Results section (Page 22, Lines 394 and 401) of the revised manuscript.

- **Comparison of assay LOD with real-time RT-PCR.** Real-time RT-PCR has so far been the most widely used molecular method to detect CCHFV genomic sequences. A number of commercial and in-house assays have been developed in the recent years (Reviewed in Mazzola *et al* 2019, PMID: 30899574). The LODs of these assays largely fall in a range of 5 – 1000 cp/reaction and are frequently seen around 10 cp/reaction (Sas *et al* 2018, PMID: 29408661; Atkinson *et al* 2012, PMID: 22217175; Garrison *et al* 2007, PMID: 17827370;  [Jääskeläinen](https://pubmed.ncbi.nlm.nih.gov/?sort=date&size=200&term=J%C3%A4%C3%A4skel%C3%A4inen+AJ&cauthor_id=25514124) *et al* 2014, PMID: 25514124). The LOD of the degenerate-sequence CRISPR assay, at roughly 25 cp/reaction (Fig 3H), appeared to be comparable to those of real-time RT-PCR assays. We have included this comparison in the Discussion of the revised manuscript (Page 28, Lines 532-538).

**Conclusions**

*I have no concerns about the Conclusions. The limitations are clearly stated, and the overall study implications are not exaggerated. The public health relevance is addressed.*

**Editorial and Data Presentation Modifications?**

*- Table 3: add “virus” and the abbreviation for consistency.*

*- Please check over the use of virus names and either spell them out or use the abbreviation. The use throughout the manuscript is inconsistent.*

**Our response:** We thank the Reviewer for noting the inconsistency in virus names. In the revised manuscript, we have added “virus” to Table 3, and have used the standard full name of those viruses consistently throughout the manuscript.

**Summary and General Comments**

*The manuscript by Li and colleagues is an interesting study applying CRISPR to the detection of genetically diverse pathogens. This proof of concept study describes the incorporation of degenerate nucleotides into CRISPR-based diagnostic assays. The manuscript is well written, and stating this is a proof of concept study and highlighting limitations is greatly appreciated! The manuscript would be strengthened if some kind of a comparator assay were included in the assay LODs. This would provide the reader with an idea about how the sensitivity compares to real-time RT-PCR or something similar.*

**Our response:** We thank the Reviewer for recognizing the significance and quality of our work. We also appreciate the very helpful suggestion of assay comparison. In response to the suggestion, we have compared the LOD of our assay with those of real-time RT-PCR for CCHFV that are well documented in published studies. The comparison is described above and has been added to the Discussion of the revised manuscript (Page 28, Lines 532-538).
